# Packing of Fruit Fly Parasitoids for Augmentative Releases

**DOI:** 10.3390/insects3030889

**Published:** 2012-09-20

**Authors:** Pablo Montoya, Jorge Cancino, Lía Ruiz

**Affiliations:** Programa Moscafrut SAGARPA-IICA, Camino a los Cacahotales S/N, CP 30680, Metapa de Domínguez, Chiapas, México

**Keywords:** Augmentative Biological Control, chilling adult technique, wide-area approach

## Abstract

The successful application of Augmentative Biological Control (ABC) to control pest fruit flies (Diptera: Tephritidae) confronts two fundamental requirements: (1) the establishment of efficient mass rearing procedures for the species to be released, and (2) the development of methodologies for the packing and release of parasitoids that permit a uniform distribution and their optimal field performance under an area-wide approach. Parasitoid distributions have been performed by ground and by air with moderate results; both options face challenges that remain to be addressed. Different devices and strategies have been used for these purposes, including paper bags and the chilled adult technique, both of which are commonly used when releasing sterile flies. However, insect parasitoids have morphological and behavioral characteristics that render the application of such methodologies suboptimal. In this paper, we discuss an alternate strategy for the augmentative release of parasitoids and describe packing conditions that favor the rearing and emergence of adult parasitoids for increased field performance. We conclude that the use of ABC, including the packaging of parasitoids, requires ongoing development to ensure that this technology remains a viable and effective control technique for pest fruit flies.

## 1. Introduction

Augmentative Biological Control (ABC) is defined as the strategy by which "a very large number of natural enemies are reared and released in critical periods for the suppression of pest populations in the short term" [1]. Some authors [2,3,4,5] suggest that this strategy can, under specific circumstances, be successfully integrated with the Sterile Insect Technique (SIT) in programs developed at the regional level.

Many integrated pest management (IPM) programs are focused on a sustainable approach to control pests, in order to mitigate the adverse effects commonly associated with the indiscriminate use of pesticides [6]. Several fruit fly management programs have incorporated ABC as a viable strategy in the integrated management of fruit flies, particularly in marginal areas that harbor a high density of alternate hosts and where the implementation of chemical control is not socially, ecologically and/or economically appropriate [7]. The greatest strength of ABC is believed to be in combination with the Sterile Insect Technique, where synergistic results are expected, resulting in higher levels of control than using either technique alone [2,3,8,9]. 

The ABC method may be a suitable alternative to solve certain limitations associated with the application of classical biological control [10,11]. These limitations include low levels of biodiversity in most agricultural systems including fruit orchards, low environmental stability, the gap between the presence of insect pests and their natural enemies in agroecosystems, and the lack of refuge as result of the loss of plant biodiversity [6]. Further, fruit flies also have greater fecundity (*i.e.*, a higher intrinsic rate of growth) and dispersal abilities than their parasitoids [12,13]. The effectiveness of parasitoids to suppress insect pests could be compromised by these factors [14,15], however the ABC approach employs the dispersal of large numbers of parasitoids mainly via human facilitation.

For ABC to be effective against fruit flies, parasitoid releases must be performed in isolated ecosystems or in areas large enough to minimize the effects of migration of the pest and parasitoids [3]. These conditions are frequently not readily available, either because there is not adequate funding to cover large areas, or because field isolation does not exist under the conditions theoretically required [3]. However, there are specific circumstances where the use of parasitoids may become an important component of the integrated management of these pests, including a) organic fruit growing areas, where the conventional use of chemicals is severely restricted; (b) canyons and other inaccessible areas where there are important quantities of host fruits; (c) areas and seasons with high rainfall, where chemical control would be inefficient by air or ground; and d) marginal areas (*i.e.*, backyard host trees/orchards) where producers may not implement control measures [16]. 

Successful application of the ABC method needs to meet several requirements. The first is the establishment of mass rearing of the appropriated species to be released, which should provide highly competitive individuals at reasonable costs [6,10]. Other fundamental requirements include the development of methodologies for packaging and release of parasitoids in the field, which should ensure the maintenance of their attributes and their optimal performance and generate an uniform release density under an area-wide approach. In this paper, we aim to highlight alternative methods for the packing and release of parasitoids for augmentative release purposes and to discuss the advantages and disadvantages of such methodologies. 

## 2. Devices used for packing and release parasitoids

### 2.1. Ground Releases

Several devices have been used for packing parasitized pupa and the release of adult parasitoids in the field for fruit fly control. The ideal container for packing fruit fly parasitoids must provide adequate access to water and food supplies and optimal conditions to ensure the high emergence and copula of adults, enhanced longevity and fecundity and a high capacity for dispersal. These elements are basic requirements for efficient parasitoid host searching behavior [17]. 

*Diachasmimorpha tryoni* (Cameron) (Hymenoptera: Braconidae) was released against *Ceratitis capitata* (Wiedemann) (Diptera: Tephritidae) in Hawaii using a 3.8 litre plastic container, 14 cm deep × 20 cm diameter, with approximately 20 holes, 1.5 cm in diameter around the wall circumference and 2 cm below the rim to allow the escape of adult parasitoids. Paper bags holding 20 g of parasitized pupa (≈ 245 puparia per gram) were placed in the containers at weekly intervals when parasitoids were required [4,9]. This type of container was also used to release *Diachasmimorpha longicaudata* (Ashmead) (Hymenoptera: Braconidae) against *Anastrepha suspensa* (Loew) (Diptera: Tephritidae) in Florida [5], but instead of paper bags, a variable number of plastic cups (1–6) containing up 100 ml of parasitized pupa were used, which, in this case, were previously irradiated to prevent fly emergence. In both studies, the containers were hung in host trees the day before expected adult emergence; males emerged first, and females emerged approximately 2 days later. Both authors [4,5] reported a successful release of parasitoids with high levels of fruit fly parasitism.

Some action programs have adopted technologies developed for sterile fruit fly releases (see [7,18]) without first assessing the appropriateness of their use for parasitoids. For instance, the use of paper bags for packing sterile flies prevailed for many years in action programs [19]; however, these bags represented a serious problem in the packing of *D. longicaudata* because, unlike flies, parasitoids have mandibles that can tear the bags and allow them to escape, causing a significant loss in the numbers required for adequate field control [20]. In Mexico, this motivated the evaluation of different types of bags in the packing of *D. longicaudata* (*i.e.*, paper bags of 80 g thick, paper bag of 50 g thick, or waxed glassine paper bag) using different densities of pupae per bag (500, 1,000, 2,000 and 4,000) prior to emergence [20]. It was observed that in paper bags, the loss of parasitoids varied from 23.3 to 56.4% and that only waxed glassine bags appropriately prevented the escape of parasitoids; however, waxed glassine bags also presented three problems: (1) poor biodegradation in the field, (2) low availability in the market, and (3) higher costs. Resultingly, alternative release methods were sought (see section 3.0).

For parasitoid species that require long pre-oviposition periods, the design of an appropriate packing container could be more elusive. For instance, *Fopius arisanus* (Sonan) (Hymenoptera: Braconidae), an egg parasitoid that successfully attacks *Bactrocera* spp. and *C. capitata* in Hawaii [21], requires a ten-day pre-mating period prior to being released and air flow to avoid the excessive accumulation of mating pheromones [22,23]. The conditions (among others) make this parasitoid species highly expensive to use for mass releases [23,24,25]. 

### 2.2. Aerial Releases—Chilled Adult Technique

The chilled adult release method for sterile fruit flies is the most common release system because it favors a more homogenous distribution in the field and avoids the dissemination of undesirable residue (*i.e.*, remains of bags and pupae) [26,27]. Insects are packed during their pupal stage in various containers depending upon the program and include Plastic Adult Rearing Containers (PARCs) (53 cm × 32 cm × 46 cm) or sieves arranged in towers (see [28]) for their emergence and sexual maturation. Prior to release, the flies are cooled for 45 min at 3 °C to achieve a degree of immobility, known as "knockdown", allowing for better handling of the insects for aerial release [26,27]. The time which insects must remain at low temperatures can vary, depending on the distance from the packing centre to the airport, the location of the release area in the field, and the aircraft transport time to reach the target area [29]. This system is now considered successful, and it is used worldwide in various SIT programs for the suppression and eradication of pest fruit fly populations and incursions.

However, the utility of the chilled adult technique for the release of fruit fly parasitoids is undetermined because little is known about the efficiency of these methods and their effects on the performance of released parasitoids. The first report evaluating the effect of the chilling system on parasitoids was in 2000 under two alternative scenarios [30]. The first scenario involved chilling the parasitoids at 3.6 °C for 1–1.5 h and packing them in paper bags to be released by aircraft for the control of *C. capitata* on coffee plantations. Paper bags containing chilled adults were dropped from a single engine airplane at an height of ~ 100 km and a rate of 25 bags/min. The second application utilized the Auger Sterile Release Machine to release the chilled adults from an aircraft. In this study the authors collected the falling adults in specific release zones on the ground where eight technicians spaced 25m apart aspirated released parasitoids for subsequent quality control bioassays, but they failed to find evidence of damage or mortality on the released insects [31]. In a complementary study by Baeza *et al*. [30], the chilling process (60 min at 3.5 °C) did not significantly affect the fecundity and life span of *D. longicaudata*, *D. tryoni* and *Diachasmimorpha kraussii* (Fullaway) (Hymenoptera: Braconidae).

A more recent study [32] has shown that adults of *D. longicaudata* are highly sensitive to packing conditions and manipulation during the chilling process. The authors evaluated three packing densities (40,000, 20,000 and 10,000 pupae per box) in two different types of PARC: 1) A standard PARC (the same box used for packing fruit flies, with lateral windows of 28 cm × 12cm), and 2) a modified PARC with larger lateral windows (40cm × 25cm) to increase ventilation. They also evaluated three different packing densities (32,900, 16,500 and 8,500 pupae) per sieve of 66cm × 72cm × 4 cm arranged in “Guatemala” eclosion towers (see [27] for a description). When the eclosed adults reached sexual maturity (approximately 5 days after emerging), they were moved to a chilled room (3 ± 2 °C) for 45 min to achieve ‘knockdown’. The chilled adults are then collected in an aerial release machine type Mubarqui^®^ [33], from which parasitoid samples were taken to determine longevity (without and with water and food), fecundity and flight ability. Packing density aeration conditions (*i.e.*, the size of the windows for ventilation) and the chilling process exerted a significant and negative influence on longevity (with water and food) and flight percentage but not on fecundity. This work also found that the packing condition and especially the chilling process [32] resulted in a notable amount of damage (30%–63% in normal PARC boxes; 32%–39% in modified PARC boxes; 27%–54% in towers) to elements of the adult female anatomy, including antennae, wings, legs and ovipositor. The main factor responsible for this damage appears to be the morphology of these insects, which, unlike the fruit flies, have long antennae, legs and ovipositors protruding from the body that become entangled among individuals when they are packed by the thousands (or millions) inside the release boxes. However, this morphological damage apparently had less of an effect than was initially thought. In this study, parasitoids were also evaluated for their searching capacity [32] (Table 1). No significant differences were observed among parasitoids derived from the chilled adult technique, parasitoids with artificially imposed damage to their antennae (a single antennae was cut mechanically and undamaged (control) parasitoids.

The deleterious effects observed when using the chilled adult technique on adult parasitoids are likely due primarily to two reasons: (1) the stress experienced by the insects under high packing densities, as has been noted for fruit flies [34], and (2) the effects of the chilling and release procedures because the frozen temperatures form crystals that increase the risk of breaking the filamentous structures under crowding conditions [35].

**Table 1:** The percentage (±SE) of *Diachasmimorpha longicaudata* females that were chilled (at 3 ± 2 °C) or unchilled and which responded to *Anastrepha ludens* infested mangoes after 10, 30, and 60 min of observation (data adapted from [32]).

	Time (min)		
Treatments	10	30	60
(1) Control	16.9 ± 3.6 a	27.8 ± 5.7 a	22.1 ± 5.2 a
(2) Artificial damage	8.4 ± 2.9 a	20.0 ± 3.2 ab	15.4 ± 3.2 a
(3) Chilling process	6.9 ± 2.8 b	12.4 ± 4.0 b	18.2 ± 5.2 a

## 3. An Alternate Strategy for Packing and Release of Parasitoids

In Mexico, D. longicaudata is mass reared on third-instar (8-day-old) A. ludens larvae, previously irradiated at 45 Gy to prevent the emergence of adult flies from any unparasitized pupae [36]. Two days before adult eclosion, the parasitized pupae are packed and sent to the required destination for field release. The Mexican campaign against fruit flies has adopted an alternate strategy for the release of parasitoids by terrestrial means, which is detailed below.

### 3.1. Modified Plastic Container

**Figure 1:**
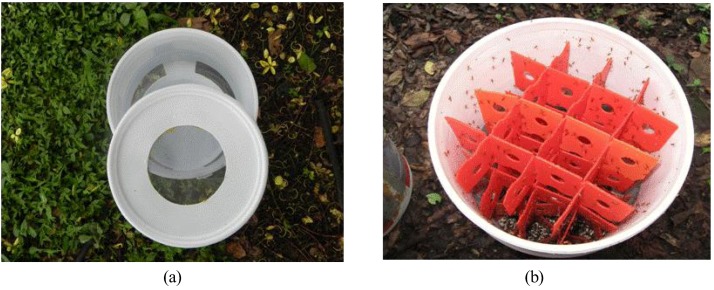
Container used for ground releases of *D. longicaudata* (**a**). Within the container are six plastic strips (24 × 8 cm) inserted to increase the surface area for parasitoids to rest (**b**).

A modified circular plastic container (20L; 30cm × 30cm) with two windows, one opposite the other, each measuring 35 cm x 11 cm, to maintain ventilation inside the container was developed to release parasitoids terrestrially (Figure 1a). The windows are covered with insect screen fiberglass, 1.5 mm thick. The lid of the container has a circular cut of 15 cm, which is covered with fiberglass mesh of the same gauge [37]. Inside the container are six alternating plastic strips measuring 24 cm × 8 cm, which increase the resting surface for the emerging parasitoids (Figure 1b). The total surface available for resting is 11,673.72 cm^2^, which can hold approximately 6,000–6,500 adult parasitoids [38]. 

### 3.2. Food for Parasitoids

Dietary requirements of eclosed adult parasitoids consist of a mixture of honey and tissue paper (17g of honey: 1g shredded paper); which has proven to be an excellent food [39]. Both ingredients are mixed in a blender or manually until the mixture acquires a thick and smooth consistency, which serves to prevent parasitoids from becoming trapped in the honey [37]. The paper-honey mixture can be stored at room temperature in covered containers for two months or under refrigerated conditions for a longer period. To reduce adult mortality, water is provided using a sponge placed on the mesh, on top of the container.

### 3.3. Packing

Parasitoids are packaged as pupae inside of the plastic container. A packing density of 10,000 pupae per bucket produces approximately 6,500 emergent adults, with a density of approximately 1 adult/2cm^2^ [38]. The honey paper mixture is partially coated on the mesh of the windows of the container or inside the container. Food is provided upon adult eclosion and parasitoids are monitored until the day of liberation. 

### 3.4. Emergence and Sexual Maturation of Parasitoids

Adults are reared in a growth room at 25 ± 1 °C and 70 ± 5% RH and a light-dark photoperiod of 12:12 h for optimal emergence. Adult emergence takes on average three days; males emerge first, and two days later, females begin to emerge. To homogenize the sexual maturity of insects, the release containers are moved to a second room at 22 ± 1 °C (same photoperiod) to facilitate copulation as it is favorable to release copulated adults [37]. The field releases take place on or near the 7th day after transportation to release destinations when most of the females have eclosed and mated [37].

### 3.5. Quality Control Evaluations

To assess the quality of eclosed adults four parameters are evaluated: adult emergence, sex ratio, longevity (survival) without water and food and flight (see [40,41,42]). In the Mexican campaign, the mean values for these parameters that are considered satisfactory are: adult eclosion, 63.2 ± 6.3%; sex ratio F: M= 2.44 ± 0.4:1; survival without water and food (females 58 ± 3.6%, males 33 ± 4.7%) at 7 d after emergence and flight (percentage of eclosed adults that flew) 84.1 ± 6.1% [43]. 

### 3.6. Release of Parasitoids in the Field

Releases are based on preliminary technical plans in which the periods and densities of release are defined according to specific conditions, including fruit phenology, the size of the release area and the host trees present. Geographical Positional Satellite (GPS) services are used to precisely locate the release areas. Containers holding eclosed parasitoids are placed in air-conditioned vehicles to move the parasitoids to the target area. This is performed in the early morning (6:00–8:00 h) to provide the best conditions under which to release the parasitoids. Once in the release area, central points are selected to open the containers and permit the escape of parasitoids.

As indicated previously, releases are focused near commercial orchards, in marginal areas (e.g., backyard orchards) with high densities of alternate host fruits, which are considered “hot points” for fly populations [7,16]. Release densities fluctuate between 1,000–2,000 adults per hectare; as parasitoids tend to aggregate in places where infested fruits are abundant (see [44]). Parasitoid releases are correlated with the FTD (Flies/Trap/Day) index [45], indicating whether the pest population is experiencing suppression. To achieve effective suppression, releases should be performed using a wide-area approach or target geographically isolated areas, ensuring all locations within an area where fruit fly host trees are present receive adequate coverage. Otherwise, surrounding fruit fly populations will re-invade the release area, nullifying any positive effect from parasitoid releases. 

The release of parasitoids using the road transportation release strategy detailed above could be a viable option, depending on several local circumstances such as topography, infrastructure (*i.e.*, adequate roads to reach the target zones), size and spatial distribution of releasing areas. In the Northwest of Mexico, terrestrial releases have been successful [46] because marginal areas with hot points for fruit fly populations are located in “island conditions” (mostly in backyard orchards), which are easily reached by ground [7]. However, the agro-ecological conditions in the southwest are completely different, making ground releases costly and inefficient. These conditions demand the development of new alternatives to better distribute parasitoids in the field.

## 4. Conclusions

Augmentative biological control is considered to be a sound strategy for fruit fly control under an area-wide basis [3,5,6,7,16,47]. However, the use of the ABC to suppress fruit fly populations requires ongoing improvements to ensure it remains as an efficient and effective control strategy. The challenges include optimization of the mass rearing procedures of current and potential natural enemies while minimizing costs [48,49], as well as the development of specific methodologies for the packing and release of parasitoids similar to those discussed here. Overcoming these challenges is essential but not simple. 
